# Comparing the skill of different reanalyses and their ensembles as predictors for daily air temperature on a glaciated mountain (Peru)

**DOI:** 10.1007/s00382-012-1501-2

**Published:** 2012-09-06

**Authors:** Marlis Hofer, Ben Marzeion, Thomas Mölg

**Affiliations:** 1 Innrain 52f, Institute of Meteorology and Geophysics, University of Innsbruck, 6020 Innsbruck, Austria; 2 Institute of Meteorology and Geophysics, University of Innsbruck, Innsbruck, Austria; 3 Chair of Climatology, Technische Universität Berlin, Berlin, Germany

**Keywords:** Reanalysis, Air temperature, Skill estimation, Glacier

## Abstract

It is well known from previous research that significant differences exist amongst reanalysis products from different institutions. Here, we compare the skill of NCEP-R (reanalyses by the National Centers for Environmental Prediction, NCEP), ERA-int (the European Centre of Medium-range Weather Forecasts Interim), JCDAS (the Japanese Meteorological Agency Climate Data Assimilation System reanalyses), MERRA (the Modern Era Retrospective-Analysis for Research and Applications by the National Aeronautics and Space Administration), CFSR (the Climate Forecast System Reanalysis by the NCEP), and ensembles thereof as predictors for daily air temperature on a high-altitude glaciated mountain site in Peru. We employ a skill estimation method especially suited for short-term, high-resolution time series. First, the predictors are preprocessed using simple linear regression models calibrated individually for each calendar month. Then, cross-validation under consideration of persistence in the time series is performed. This way, the skill of the reanalyses with focus on intra-seasonal and inter-annual variability is quantified. The most important findings are: (1) ERA-int, CFSR, and MERRA show considerably higher skill than NCEP-R and JCDAS; (2) differences in skill appear especially during dry and intermediate seasons in the Cordillera Blanca; (3) the optimum horizontal scales largely vary between the different reanalyses, and horizontal grid resolutions of the reanalyses are poor indicators of this optimum scale; and (4) using reanalysis ensembles efficiently improves the performance of individual reanalyses.

## Introduction

Even though reanalysis, by using the methods of numerical weather prediction, is the most accurate way to interpolate atmospheric data in time and space, its usefulness to document climatic trends and variability is debated (e.g., Kalnay et al. 1996; Bengtsson et al. 2004). A major source of uncertainty in reanalysis comes from errors or deficiencies in the observations needed to assimilate the model solutions towards the true atmospheric state. In particular, changes in the observing system have shown to cause artificial climate variability and trends such as the introduction of satellite data in the late 1970s, as well as changes of observation density; e.g., Trenberth et al. (2001) and Bengtsson et al. (2004). Another major source of problems includes uncertainties in the atmospheric models used to generate the background forecast for the data assimilation. Reanalysis data documentations and many other studies report about these limitations (e.g., Kalnay et al. 1996; Trenberth et al. 2001; Uppala et al. 2005; Rood and Bosilovich 2009; Chelliah et al. 2011; Dee et al. 2011).

Global reanalysis data are generated at four institutions worldwide (in cooperation with partner institutions not mentioned here for brevity): the National Centers for Environmental Prediction, NCEP (Kalnay et al. 1996; Kanamitsu et al. 2002; Saha et al. 2010); the European Centre for Medium-Range Weather Forecasts, ECMWF (Uppala et al. 2005; Dee et al. 2011); the Japan Meteorological Agency, JMA (Onogi et al. 2007); and the National Aeronautics and Space Administration NASA (Rienecker et al. 2011). An overview about all global reanalyses with availability up to present is given in Table 1 (despite the NCEP/Department of Energy reanalysis 2, Kanamitsu et al. 2002, that are also available up to present but not considered in this study). Second-generation reanalyses have profited from the increasing availability and a better treatment of the assimilated observations, from advances in computing power and modeling systems, and other lessons learned from problems in the earlier projects (e.g., Rood and Bosilovich 2009). Due to artificial jumps in the data caused by major changes in the observing system, more recent reanalyses are restricted to data-rich periods, the satellite era (i.e., from 1979 onwards). Since the beginning of the satellite era throughout the assimilation period, observations assimilated in the reanalyses have still increased tenfold (e.g., from approximately 10^6^ observations assimilated per day in 1979 to 10^7^ in 2005, Dee et al. 2011; Rienecker et al. 2011), most of this increase originating from satellite data. In the more recent reanalyses, satellite data are more efficiently used as they include direct assimilation of satellite radiances, and automated schemes for bias-corrections of radiances (Saha et al. 2010; Dee et al. 2011; Rienecker et al. 2011). With increasing computer power available, higher performance 4D-Var (4-dimensional variation analysis) became feasible for reanalysis for the first time (Dee et al. 2011). Spatial resolutions of the reanalyses largely vary from triangular truncations T62 to T382 (corresponding to horizontal grid resolutions from 2.5° to 0.5°), with 28–72 levels in the vertical (cf. Table 1). Temporal resolutions are 6-hourly or higher for all reanalyses.

**Table 1:** Overview about all global reanalyses used in this study

	NCEP-R	ERA-int	JCDAS	MERRA	CFSR
Generation	1st	2nd	2nd	2nd	2nd
Status	Operated	Operated	Operated	Operated	Operated
Period	1948-	1979-	1979-	1979-	1979-
Spatial res.	T62 L28	T255 L60	T106 L40	2/3 × 1/2 L72	T382 L64
Temporal res.	6-hourly	6-hourly	6-hourly	3-hourly	hourly
System	3D-Var	4D-Var	3D-Var	3D-Var	3D-Var
Institution	NCEP	ECMWF	JMA	NASA	NCEP

Studies exist that compare different reanalysis data in some regards. Simmons and Jones (2004) evaluate trends and low-frequency variability in surface air temperature of ERA-40 (the 45 years ECMWF reanalysis) and NCEP-R (the NCEP/NCAR reanalysis) with CRU (Climate Research Unit, Jones and Moberg 2003) data sets globally. Dessler and Davis (2010) analyze NCEP-R, ERA-40, JRA-25 (the Japanese 25-yr reanalysis), MERRA (the Modern Era Retrospective- Analysis for Research and Applications from NASA), and ERA-int (the ECMWF interim reanalysis) with regards to tropospheric humidity trends. They find artificial negative long-term trends in NCEP-R tropospheric humidity and large bias in NCEP-R tropical upper tropospheric humidity not evident in all the other reanaly ses. Bosilovich et al. (2008) show that reanalysis precipitation improves in recent systems and that ERA-40 products show reasonable skill over northern hemisphere continents, but less so in the tropical oceans, whereas JRA-25 shows good agreements in both tropical oceans and northern hemisphere continents. Trenberth et al. (2001) study the quality of ERA-15 (the 15-years ECMWF reanalyses) and NCEP-R air temperatures in the tropics, finding that ERA-15 show large discrepancies to observations due to changes in the satellite system, whereas NCEP-R show good agreement. Wang et al. (2011) find that the NCEP Climate Forecast System Reanalysis (CFSR) show improved tropical rainfall variability compared to NCEP-R and NCEP/Departement of Energy (DOE) reanalyses 2. Chelliah et al. (2011) document disagreements of the CFSR with other available reanalyses, in terms of stronger easterly trades, cooler tropospheric temperatures, and lower geopotential heights during the earlier part of the reanalysis period (1979–1998). All studies report about important differences amongst different reanalysis types.

**Fig. 1:**
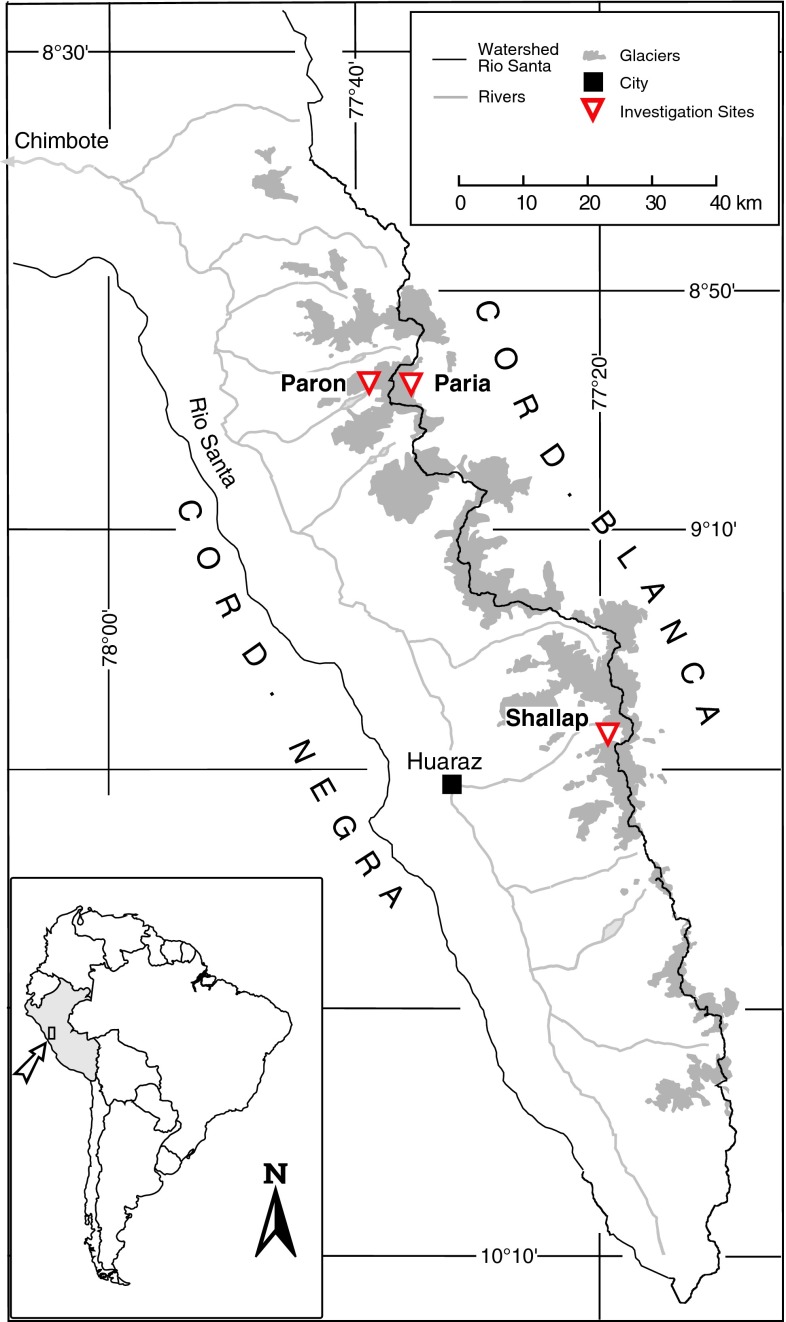
Map of the Rio Santa watershed with the Cordillera Blanca mountain range, and measurement sites (as described in the text). Also indicated is the 1990 glacier extent (*grey shaded area*; Georges 2004)

In this study we compare the skill of different reanalyses and their ensembles as predictors for site-specific, daily air temperature in the tropical Cordillera Blanca (cf. Fig. 1). The Cordillera Blanca is a glaciated mountain range in the northern Andes of Peru, harboring 25 % of all tropical glaciers with respect to surface area (Kaser and Osmaston 2002). The glaciers have heavily shaped the socioeconomic development in the extensively populated Rio Santa valley, with the occurrence of several disastrous glacial lake outburst floods and ice avalanches (Carey 2005, 2010). On the other hand, melt water from the currently shrinking glaciers (Ames 1998; Georges 2004; Silverio and Jaquet 2005) is an important water source for agriculture, households and industry in the dry season (Mark and Seltzer 2003; Kaser et al. 2003; Juen 2006; Juen et al. 2007; Kaser et al. 2010), when precipitation is extremely scarce (Niedertscheider 1990). To quantify the impacts of future climate change to glaciers in the Cordillera Blanca is thus of primary relevance. Due to the absence of long-term, high-resolution atmospheric measure ments in the Cordillera Blanca, however, longer-term, process-based assessments of the glacier-atmosphere link (e.g., Mölg et al. 2009) is problematic. Hofer et al. (2010) and Hofer (2012), by means of empirical-statistical downscaling, explore the potential of NCEP-R to provide more knowledge about past atmospheric variations in the Cordillera Blanca, with promising results. The goal of the present study is to identify the most appropriate data set, beyond NCEP-R out of all available reanalyses, for the study site and target variable. Whereas we do not claim the results being valid outside the study area or for different variables, the method presented here provides the basis for inter-comparison studies of reanalysis data, and large-scale model output in general, as predictors for different locations and target variables. In Sect. 2 we present the data sets used in this study. Section 3 provides an overview of the applied methodology. Finally, we show the results in Sect. 4 and the conclusions in Sect. 5.

## Data

The skill assessment of reanalyses in this study is based on local air temperature measurements carried out at a high-altitude site in the glaciated Cordillera Blanca mountain range (Fig. 1). In earlier studies, we focused on skill assessment of NCEP-R, using air temperature and specific humidity measurements from multiple sites in the Cordillera Blanca (Hofer et al. 2010; Hofer 2012). Air temperature measurements have shown to be rather homogenous throughout the Cordillera Blanca, and differences in NCEP-R skill with regard to different automated weather stations (AWSs) are small, with the same seasonality of skill being evident for all AWSs (not shown). In this study, it is therefore reasonable to use the time series from only one AWS. We selected the longest and most reliable, quality-controlled air temperature time series from all AWSs in the Cordillera Blanca, hereafter referred to as airt-CB. The AWS providing airt-CB is located on a moraine at 5,000 m a.s.l. (meters above sea level), corresponding to a mean air pressure of about 560 hPa, in the Paron valley (Northern Cordillera Blanca, cf. Fig. 1). airt-CB is measured with a HMP45 sensor by Väisalla in a ventilated radiation shield (described by Georges 2002), mounted at two meters above the ground. To date, airt-CB is available from 07/2006 to 07/2010. In Fig. 1, two further sites are indicated, where AWSs exist on and next to glaciers: Paria, located close to the Paron valley but east of the main divide, and Shallap in the southern Cordillera Blanca, west of the main divide (Juen 2006).

**Fig. 2:**
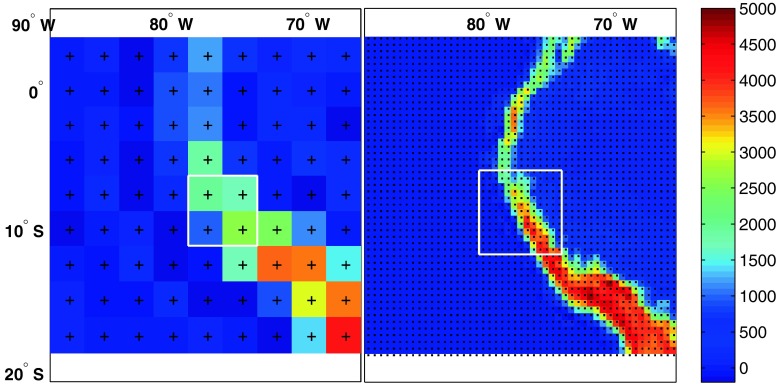
NCEP-R (*left*), and CFSR (*right*) model topographies (meters above sea level) and grid resolutions (*crosses*, and* dots*, respectively) for the South American sector considered in this study (note that both plots include the same area). The* white rectangles* indicate the optimum horizontal scales centered around the study location

In this study we consider five different reanalyses: (1) NCEP-R, (2) ERA-int, (3) JCDAS (the JMA Climate Data Assimilation System reanalyses), (4) MERRA and (5) CFSR (see Table 1 for details about the data sets). These are, apart from the NCEP/DOE reanalysis 2 (Kanamitsu et al. 2002), all available reanalysis data that cover the period of available measurements in this study, provided up to the present at the respective institutions NCEP, ECMWF, JMA and NASA. All data are downloaded on their native spatial grids, in an area extending from 5°N to 20°S and 90°W to 65°W (area displayed in Fig. 2), and from the 400 to 700 hPa levels.

## The method

We apply a method of skill assessment designed specifically for model inter-comparisons when only short, but high-resolution observational time series are available. The simple procedure is comprehensibly outlined below, in order to allow for easy transference to different cases (e.g., in terms of sites, or predictors).

The reanalysis model predictors (*x*) are first preprocessed using simple linear regression models, ($\hat{y}$), calibrated separately for each calendar month, *m*:

1 $$ y_{\rm s}(t_{\rm m})=a_{\rm m} \cdot x_{\rm s}(t_{\rm m})+\epsilon(t_{\rm m}) \quad m=1,\ldots,12, $$

where *t* is the time variable (omitted in the subsequent equations for the sake of brevity), *y* are the observations, or target variables (here, daily means of airt-CB), index *s* denotes the variables being standardized, and ε is the model error, obtained as the difference between *y* and $\hat{y}$

2 $$ \epsilon=y_{\rm s}-\hat{y}_{\rm s}. $$

From Eqs. 1 and 2 it is apparent that

3 $$ \hat{y}_{\rm s}=a_{\rm m} \cdot x_{\rm s}. $$

It can be shown that the regression parameter, *a*_m_, is exactly the correlation coefficient here (Von Storch and Zwiers 2001). Note that *a*_m_ consists of twelve values (one for each calendar month).

Then, skill estimation is repeated for $\hat{y}_{\rm s}(x)$ based on predictors *x* from all five reanalysis data assessed in this study, NCEP-R, ERA-int, JCDAS, MERRA and CFSR (and ensembles thereof). Evaluating $\hat{y}_{\rm s}(x)$, as defined above, rather than the untransformed predictors, *x*, can be viewed as essential data preprocessing step especially useful for short-term observational time series, *y*, for the following reasons. (1) The skill assessment is focused on performance of the reanalysis predictors in capturing intra-seasonal, and inter-annual variations, rather than the seasonal cycle. This is important because seasonal variations are generally larger than inter-annual and intra-seasonal variability and would otherwise dominate the results. When long enough data series are available, the problem can be avoided also by subtracting the climatological seasonal cycle from the time series (e.g. Madden 1976). Yet by subtracting the climatological seasonal cycle, seasonally varying performances of predictors are not accounted for and by contrast here, the performances of the predictors are quantified for each month individually. (2) The skill assessment does not penalize for differences in monthly means and variances between reanalyses and observations. This allows for more general inter-comparisons of predictor variables from different levels (as performed in this study), or with different physical units.

The skill estimation is based on a modification of leave-one-out cross-validation that accounts for autocorrelation in the daily time series and therefore guarantees complete independence between training and test data. The skill score, *SS*_clim_, can be calculated (Wilks 2006)

4 $$ SS_{\rm {clim}}=1-{\frac{mse}{mse_{\rm {clim}}}} $$

based on *mse*, the mean squared error

5 $$ mse={\frac{1}{n_{\rm cv}}}\cdot\sum{\epsilon_{\rm cv}^2} \quad\quad \epsilon_{\rm cv}=y_{\rm s,v}-\hat{y}_{\rm s,v}(x_{\rm s,v}) $$

and *mse*_clim_, the mean squared error of the reference forecast, here a cross-validation-based estimate of the sample variance, as follows

6 $$ mse_{\rm clim}={\frac{1}{n_{\rm cv}}}\cdot\sum{(y_{\rm s,v}-\hat{y}_{\rm r})^2}\quad\quad \hat{y}_{\rm r}:=\overline{y_{\rm s,c}}. $$

Above, ε_cv_ is the difference between independent observation *y*_s,v_ and model, $\hat{y}_{\rm s,v}(x_{\rm s,v})$ (*v* means validation), obtained for the cross-validation repetition *cv*, with *cv* = 1, …, *n*_cv_ (*n*_cv_ is the number of cross-validation repetitions). $\hat{y}_{\rm r}$ is defined as the mean of all observations used for the model training, *y*_s,c_ (c for calibration). *mse*_clim_ is the variance of *y*_s_, estimated based on cross-validation, and thus not exactly one, but slightly larger (i.e., involving the difference of the independent observation *y*_s,v_ to the mean of the observations used in the model training *y*_s,c_). *SS*_clim_, as defined above, is also known as reduction of variance, because the quotient being subtracted is the average squared error divided by the climatological variance (here estimated by cross-validation). *SS*_clim_ is a measure of the covariance between modelled and observed time series (similar to the squared correlation coefficient, *r* ^2^), but accounts further for errors in estimating the variance (reliability of the forecast), and for model biases (see Murphy 1988; Wilks 2006). In this regard *SS*_clim_ is the more accurate goodness-of-fit measure than *r* ^2^ (i.e., lower than *r* ^2^).

Regarding the choice of predictor variables, downscaling studies generally suggest to use a combination of circulation-based, and radiation-based predictors for air temperature predictands (e.g., Huth 2004). Yet, specific recommendations vary broadly amongst the different studies (e.g., Von Storch 1999; Wilby and Wigley 2000), and the definite choice of optimum predictor variables requires data-based assessments. In this study, we suggest to relate the same physical predictor and target variables (i.e., air temperature for the predictand air temperature) as intuitive, a priori choice. A priori selections are based on information outside the data (i.e., prior to data analysis), and therefore provide a more appropriate basis for model inter-comparison studies than data-based selections. The a priori assumption here is that the best model shows the highest skill in representing the same variable, because it is closer to reality - an assumption that applies similarly for different sites and variables. Still, we emphasize that this is not necessarily the best predictor choice for individual models.

To identify the optimum downscaling domain for each of the five reanalyses, we conduct a systematic assessment of model skill as a function of spatial averaging. Grid point averaging of atmospheric models to obtain higher skill predictors can be considered as a compromise between minimizing numerical model errors related to single grid point data (Grotch and MacCracken 1991; Willamson and Laprise 2000; Räisänen and Ylhäisi 2011) and loosing climate information at the minimum model scale (i.e., the distance of two neighboring grid points). Due to the pronounced spatial homogeneity of synoptic forcing in the region (Garreaud et al. 2003), we suspect the latter effect being less dominant for the predictand air temperature in the Cordillera Blanca than it might be for other sites. After determining the optimum scale for each reanalysis, their performances relative to each other are compared at their individual optimum scales.

The optimum scale analysis, where we distinguish between horizontal and vertical domain extensions, is done as follows. For each reanalysis, the grid point located closest to the study site is identified and the skill assessment is conducted for the single grid point predictor, as explained above. Thereafter, the horizontal domain of averaging is increased consecutively by the minimum scale of each reanalysis (the minimum scale is 2.5° for NCEP-R, 0.72° for ERA-int, 1.25° for JCDAS, and 0.5° for MERRA and CFSR, cf. Table 1) and the skill assessment is repeated for each domain. Then the two closest vertical levels are added, and the analysis is started over by first considering only the horizontally closest grid point (now in more levels) and then consecutively increasing the horizontal area (as it was done for the single level domain before). Table 2 shows an overview of all horizontal domain-vertical level combinations considered in this study. Number of grid points, scales of the horizontal domains, as well as vertical levels change for the different reanalyses because of their different spatial resolutions. For MERRA, in particular, the number of grid points *n*_gp_ for domain *n* is not like for the other reanalyses *n*_gp_ = *n* ^2^, because latitudinal and longitudinal grid resolutions of MERRA are not the same (1/2° and 2/3°, respectively). The data-based optimum scale-analysis gives important insight to the performance of the individual reanalyses: in particular, (1) discrepancy between minimum scale and optimum scale indicates errors related to numerical noise, or remote grid point predictors playing a more important role than nearby ones; and in general (2), the larger the optimum scale, the lower the performance of the reanalysis system can be assumed.

**Table 2:** Vertical levels (hPa; upper row) and horizontal domains (lower row) considered for each reanalysis

	NCEP-R	ERA-int	JCDAS	MERRA	CFSR
Vert.levels	*sl*- 400:100:700 *ml*- 500:100:600 400:100:700	*sl*- 400:50:700 *ml*- 500:50:600 450:50:650 400:50:700	(same as for NCEP-R)	(same as for ERA-int)	(same as for ERA-int)
Hor.domains	1gp (2.5°)	1gp (0.72°)	1gp (1.25°)	1gp (0.5°)	1gp (0.5°)
	4gp (5°)	4gp (1.44°)	4gp (2.5°)	2gp (1° × 0.5°)	4gp (1°)
	9gp (7.5°)	9gp (2.16°)	9gp (4.75°)	9gp (1.5.°)	9gp (1.5°)
	16gp (10°)$\ldots$	16gp (2.88°) $\ldots$	16gp $(6^{\circ}) \ldots$	12gp (2° × 1.5.°) $\ldots$	16gp (2°) $\ldots$

*sl* means single level, and *ml* multiple levels. In the case of *sl*, all listed levels (*a*:*b*:*c*; i.e., all levels within *a* and *c* in intervals of size *b*) are considered individually. In the case of *ml*, averages over the listed levels are considered. In terms of horizontal domain, in front of gp is the number of grid points considered in each domain, the value in brackets indicates the scale of the respective domain. The horizontal domains are increased until a maximum scale of 25°. In our analysis, each of the horizontal domains is combined with each of the vertical level combinations

In the optimum domain analysis of this study we disregard the assessment of domains not centered around the study site (as proposed, e.g., by Brinkmann 2002). Here we assume that the best models also show the highest skill in the vicinity of the study site, because they are closer to reality. Similarly, we expect the optimum domain to be smallest for the best model. Again, we emphasize that this is not necessarily the best choice for each individual case (or model), but a reasonable starting point for predictor or model inter-comparisons. Note furthermore that this assumption is more problematic for precipitation downscaling (e.g., Maraun et al. 2010), because precipitation is associated with larger model uncertainty, and downscaling relationships are generally more complex (i.e., including multiple predictors and non-linear relationships) than for air temperature predictands. For example, Wilby and Wigley (2000) find that optimum predictor domains for precipitation downscaling are not necessarily located immediately above the target location, depending on the predictor variables applied. Sauter and Venema (2011) identify asymmetric, not rectangular optimum domains that are physically interpretable in terms of atmospheric processes. For empirical-statistical downscaling studies that consider remote grid point information, we recommend using principal component (PC) analysis (e.g., Hannachi et al. 2008; Schubert and Henderson-Sellers 1997; Huth 2004; Hofer et al. 2010), as PC analysis effectively separates important atmospheric modes from noise in large data sets.

## Results and discussion

### Optimum scale analysis

**Fig. 3:**
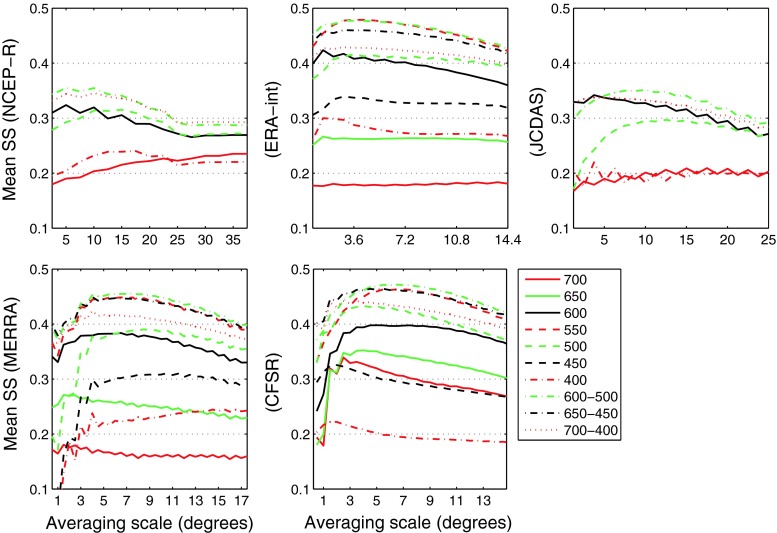
Values of *SS*_clim_ averaged over all calendar months (mean *SS*) for different vertical levels and combinations (*different colors* and line properties) and increasing horizontal domains (from* left* to* right*), for the five reanalyses considered as predictors. Please note that the scale on the abscissa changes for the different reanalyses, because of the different grid point spacings, whereas the scale on the ordinate is kept fix

Figure 3 shows mean values of *SS*_clim_ (i.e., the twelve values of *SS*_clim_ obtained for each calendar month averaged) for increasing spatial domains in different vertical levels and level combinations, for each of the predictors NCEP-R, ERA-int, JCDAS, MERRA and CFSR. For all reanalyses, mean *SS*_clim_ values increase rapidly with increasing domain scale until reaching a maximum, and then decrease slowly. In particular for ERA-int, JCDAS, MERRA and CFSR the slopes of the curves are often steeper to the left of the maxima, than to the right (cf. Fig. 3). This indicates, most notably, that by overestimating the optimum domain size by a certain scale interval, less information is lost, than by underestimating the optimum domain by this same interval (cf. abscissa in Fig. 3). Concerning the skill of the different vertical levels considered here, generally the levels close to the study site (i.e. from 500 to 600 hPa, since AWS-CB is situated at 560 hPa) show higher skill than the levels farther below or above (i.e., >600 or <500 hPa). For all reanalyses despite ERA-int, the highest mean *SS*_clim_ occurs for the 600–500 hPa multiple level averages. The highest mean *SS*_clim_ of ERA-int results for the single level domain at 550 hPa.

In the case of NCEP-R, the maximum skill is found at a scale of 5° (notably only *n*_gp_ = 4 grid points). The respective optimum scale of ERA-int is 2.88° (thus including *n*_gp_ = 16 grid points). For JCDAS, with 8.75° (*n*_gp_ = 49), a considerably larger optimum scale results. Very similar patterns of skill are evident for MERRA and CFSR, with the highest skill found at scales of 7.3° (MERRA), and 6.5° (CFSR). Because of the high horizontal resolutions of both MERRA and CFSR, this includes by far the largest amount of grid points to be averaged compared to the other reanalyses (i.e., *n*_gp_ = 154 for MERRA, and 169 for CFSR, respectively), pointing to considerable uncertainty (e.g., related to numerical noise) in MERRA and CFSR single grid point data. To sum up, for all reanalyses the optimum domain is achieved with data from the pressure levels located close to the study site. However in terms of optimum horizontal scale, or optimum amount of grid points to be averaged, respectively, results widely vary for the different reanalyses from 2.88° (ERA-int) to 8.75° (JCDAS), and from *n*_gp_ = 4 (NCEP-R) to *n*_gp_ = 169 (CFSR). Results in Table 4 are discussed further in the next section.

To visualize the relation between grid resolutions and optimum scales, the optimum horizontal domains of NCEP-R and CFSR (the first and second generation reanalyses by the NCEP, and at the same time the reanalyses with the lowest, and highest grid resolutions, respectively) are shown in Fig. 2, along with their model topographies (for the South American sector considered in this study). The large difference between the spatial resolutions of NCEP-R and CFSR is clearly evident from Fig. 2. The Cordillera Blanca is located between only 1,000 and 2,000 m a.s.l. in the NCEP-R topography, whereas it reaches much more realistic elevations of higher than 4,000 m a.s.l. in the CFSR (for comparison, peaks in the Cordillera Blanca reach up to almost 7,000 m a.s.l.). Yet the optimum horizontal domains of the coarse NCEP-R, and the fine-resolution CFSR are almost of the same size-being, in fact, even smaller for the coarse-scale NCEP-R.

**Fig. 4:**
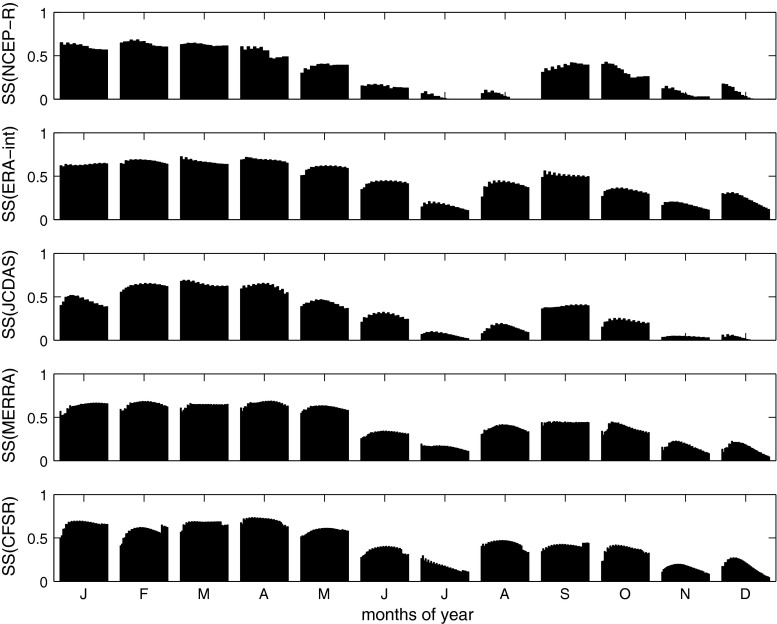
Values of *SS*_clim_ for different months, and for increasing horizontal domains (within each month increasing from* left* to* right*) in the vertical level that shows the highest values of *SS*_clim_ averaged over all calendar months, for NCEP-R (500–600 hPa,* top*), ERA-int (550 hPa,* second plot*), JCDAS (500–600 hPa,* third plot*), and MERRA (500–600 hPa,* bottom*). Note that the domains are increasing from* left* to* right*, i.e., the* first bar* refers to domain one, the* second bar* to domain two, etc. (for details about domain definitions the reader is refered to the text)

Figure 4 shows values of *SS*_clim_, at a monthly resolution, for increasing horizontal domains at the respective vertical levels for which the highest mean *SS*_clim_ occurs for each of the reanalyses. In Fig. 4, the optimum horizontal domain size is not identified as clearly, as by using mean *SS*_clim_ values as a measure (Fig. 3), because the optimum scales differ for different months. In particular for some months, values of *SS*_clim_ increase and decrease consecutively with increasing domain size. This square wave pattern on top of some bars can be explained by the geometry of the optimum domain analysis. In particular, the horizontal domains are increased by adding grid points to either western or eastern, and southern or northern sides of the domains, and in the following step, the domains are increased by adding grid points to the respective opposite sides. The pattern of consecutive increase/decrease of *SS*_clim_ then results because grid points from one direction contain more information relevant to the local-scale data than grid points from the opposite direction. This indicates that horizontal domains arranged symmetrically around the study site are not necessarily the optimum domains for downscaling, but shifting the domain towards synoptically more important regions can increase the skill (as suggested for precipitation downscaling also by Wilby and Wigley 2000; Brinkmann 2002; Sauter and Venema 2011). These optimum domain asymmetries can be expected to vary seasonally, since the square wave pattern reverses several times throughout the year (Fig. 4).

Table 3 gives a summary of months, where increases (decreases) of skill for north or south (combined with west or east) extensions occur, for all reanalyses (note that for MERRA the analysis is shown only for north or south extensions, because, due to the MERRA grid point geometry, west and east extensions occur simultaneously). The different reanalyses show the same square wave patterns for the same months. In particular with northward extensions, *SS*_clim_ values of all reanalyses increase in January, of most reanalyses in March, April, June and October, and of some reanalyses in February and December. With southward extensions, values of *SS*_clim_ of all reanalyses, despite MERRA, increase in July, August, and September. This is in some respects in accordance with the findings of Georges (2005), who performs a seasonal analysis of the tropospheric flow in several levels in the Cordillera Blanca. Even if Georges (2005) identifies northeasterly flow prevailing all year round, he finds that during humid conditions (especially January to March) the flow is more northerly than during dry conditions (June–August).

**Table 3:** List of months (January 1, February 2, $\ldots$) for which increases (+) of *SS*_clim_ for domain extensions towards southeast (SE) or northwest (NW) occur (for JCDAS southwest (SW) and northeast (NE) extensions, for MERRA south (S) or north (N) extensions). Increases for extensions towards SE on the same time imply decreases for extensions towards NW (and likewise for other directions)

	NCEP-R	ERA-int	JCDAS	MERRA	CFSR
+SE(NCEP-R,ERA-int,CFSR)	5,7,8,9,10,11	7,8,9	5,7,8,9	–	5,7,8,9
+SW(JCDAS)					
+S(MERRA)					
+NW(NCEP-R,ERA-int,CFSR)	1,4,6	1,3,12	1,2,3,4,10	1,3,4,6,10	1,2,3,4,6,10,12
+NE(JCDAS)					
+N(MERRA)					

Table 4 shows a summary of the optimum scale analysis for all reanalyses. NCEP-R, most notably, show the smallest amount of grid points to be averaged for the optimum domain of all reanalyses (4 grid points in 2 levels, thus 8 grid points in total), and therefore the optimum scale is comparably small, even if the minimum scale of NCEP-R is the largest of all reanalyses. ERA-int is the only reanalysis where single-level data show the highest skill (i.e., the level located closest to the study site) and the optimum domain of ERA-int comprises relatively few grid points (16 in total). The resulting optimum scale for ERA-int is in fact the smallest of all reanalyses, with 2.88°. In the case of JCDAS, more grid points to be averaged are required for the optimum domain, resulting the largest optimum scale of all reanalyses with 8.75°. For MERRA and CFSR the largest amount of grid points to be averaged compose the optimum domain (462 and 507, respectively).

**Table 4:** Results of the optimum domain analysis for all reanalyses: (1) skill scores (*SS*_clim_ averaged over all months, and in brackets the month where the maximum of *SS*_clim_ occurs and the maximum value), (2) amount of grid points included in the optimum domain (total number, and in brackets the amount of grid points in the latitudinal × longitudinal × vertical direction), (3) horizontal scale of the optimum domain in degrees, and (4) optimum pressure levels to be averaged (hPa)

	NCEP-R	ERA-int	JCDAS	MERRA	CFSR
(1)	0.36 (Feb:0.66)	0.48 (Apr:0.71)	0.35 (Mar:0.66)	0.46 (Feb:0.67)	0.47 (Apr:0.73)
(2)	8 (2 × 2 × 2)	16 (4 × 4 × 1)	49 (7 × 7 × 2)	462 (14 × 11 × 3)	507 (13 × 13 × 3)
(3)	5 × 5	2.88 × 2.88	8.75 × 8.75	7 × 7.3	6.5 × 6.5
(4)	500,600	550	500,600	500,550,600	500,550,600

### Comparing the skill of the different reanalyses and their ensembles

**Fig. 5:**
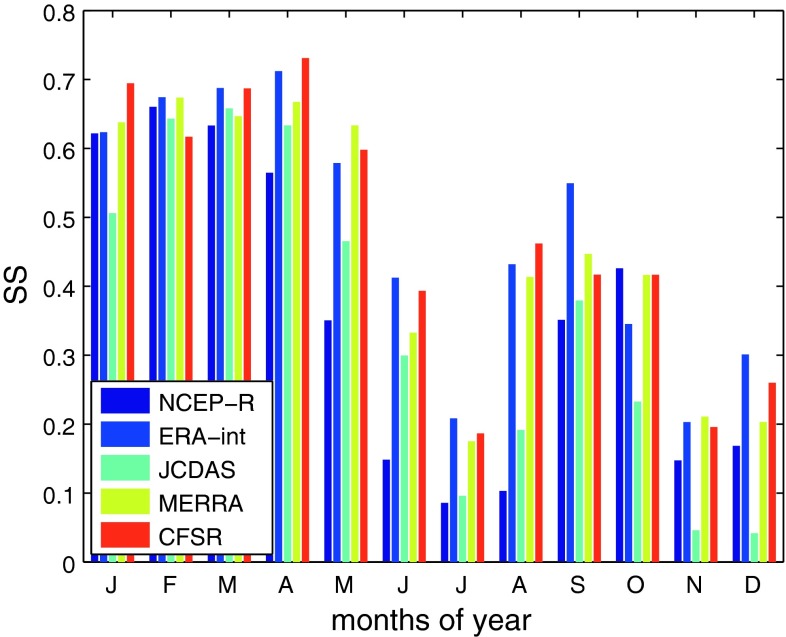
Values of *SS*_clim_ for each calendar month and the different reanalyses (*different colors* of the* bars*) considered at their respective optimum domains

Table 4 and Fig. 5 summarize the performances of the five reanalyses at their individual optimum spatial domains determined by the maximum of *SS*_clim_ averaged over all months. Of all reanalyses, ERA-int show the highest skill (mean *SS*_clim_ = 0.48). Whereas both MERRA and CFSR show comparably high skill like ERA-int (mean values of *SS*_clim_ are 0.46 and 0.47, respectively), NCEP-R and JCDAS show considerably lower skill (mean *SS*_clim_ are 0.36, and 0.35, respectively). More precisely in terms of time of year, the highest values of *SS*_clim_ result in February (for NCEP-R and MERRA), April (for ERA-int and CFSR), and March (for JCDAS); mainly wet season months in the Cordillera Blanca (for the definitions of climatological seasons in the Cordillera Blanca, please see Niedertscheider 1990). In April, CFSR achieve the highest value of *SS*_clim_ of all reanalyses in all months, with *SS*_clim_ = 0.73. A second maximum of *SS*_clim_ occurs, for all reanalyses, in the transitional period from dry to wet season, i.e. September–October. The lowest values of *SS*_clim_ appear in the core dry season (especially July), and in the intermediate months November to December. Whereas NCEP-R and JCDAS show comparably high skill like the other reanalyses during the wet season, large differences in performance appear particularly for dry season months (especially August, cf. Fig. 5). We conclude that high values of *SS*_clim_ of all reanalyses point to spatially homogenous synoptic forcing of air temperature fluctuations in the region, well represented by the reanalyses, during the respective months. By contrast, low values of *SS*_clim_ in dry season months indicate that variability must be dominated by small-scale processes (represented to some extent by the higher-resolution reanalyses—ERA-int, MERRA, and CFSR, and less so by the lower resolution reanalyses-NCEP-R and JCDAS) with the generally weaker synoptic forcing having almost no impacts (as discussed also by Hofer 2012).

**Fig. 6:**
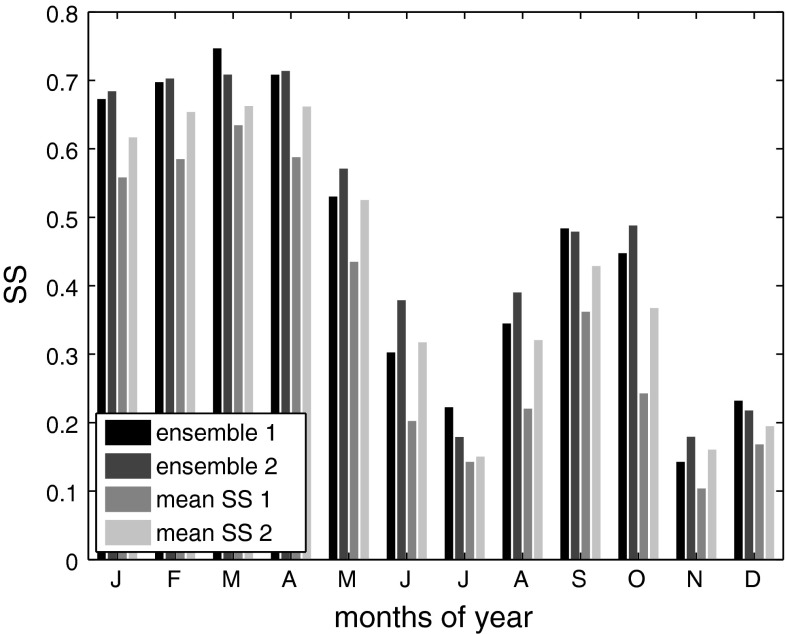
Monthly values of *SS*_clim_ for the two ensembles (ensemble 1 is the mean of all five reanalyses’ single grid point data, and ensemble 2 is the mean of the reanalyses considered at their optimum spatial domains), as well as the average of *SS*_clim_ of the five reanalyses’ single grid point data (mean *SS* 1), and the average of *SS*_clim_ of the reanalyses considered at their optimum spatial domains (mean *SS* 2;* shadings* from dark to light)

Figure 6 shows values of *SS*_clim_ of two different ensembles of the reanalyses for each month of the year. Ensemble 1 in Fig. 6 is the average of the time series from the grid points closest to the study location of each reanalysis (thus, the average of the time series of five grid points in total). Ensemble 2 is the average of the reanalyses considered at their optimum domains (thus 8 + 16 + 49 + 462 + 507 = 1,042 grid points in total). For comparison, the averages of monthly *SS*_clim_ of data from single grid points of all reanalyses (mean *SS*_clim_ 1), and the averages of monthly *SS*_clim_ of the reanalyses considered at their optimum domains (mean *SS*_clim_ 2) are shown. Note that the ensemble time series are preprocessed and skill estimation is performed similarly as for the individual, single grid point and domain averaged reanalyses (as described in Sect. 3). As evident from Fig. 6, the skill of the ensembles is generally higher than the average skill of the reanalyses considered individually. As can be expected, the skill of ensemble 2 is higher than the skill of ensemble 1 for almost all months. However, the differences are small (the values of *SS*_clim_ averaged over all calendar months are 0.46 for ensemble 1, and 0.47 for ensemble 2, respectively; for comparison *SS*_clim_ averaged over all calendar months and reanalyses is 0.35 for single grid point predictors, and 0.42 for the reanalyses considered at their optimum spatial domain). This indicates that by averaging data from different reanalyses, errors are eliminated very efficiently, in a way that it makes no large difference whether single grid point data or data from the optimum domains of each reanalysis are used in the ensembles. Spatial correlation of numerical noise is a possible reason for the large optimum domains of individual reanalyses. Even if the skill of ERA-int considered at their optimum spatial domain (mean *SS*_clim_ = 0.48) is slightly higher than the skill of the reanalysis ensembles, the use of ensembles can be advantageous, because (1) it is not necessary to determine the best reanalysis product, and its optimum scale for each specific case, and (2) less data needs to be averaged for obtaining almost the same results (e.g., in this study, 5 versus at least 16 time series).

The considerably higher skill of ERA-int, MERRA and CFSR, compared to NCEP-R and JCDAS, can be explained by several aspects. Higher-resolution topographies and accordingly physical processes resolved represent one probable reason for the higher performances of ERA-int, MERRA and CFSR (all with spatial resolutions higher than 1°), against NCEP-R and JCDAS (with spatial resolutions lower than 1°), even if this is not evident from the differences in the respective optimum scales as analyzed here (cf. Fig. 2). In terms of assimilation system, ERA-int is the only reanalysis based on the high performance 4D-Var. 4D-Var is considered a major step forward from the previous reanalyses generated at the ECMWF, since it allows for the more effective use of observations (Dee et al. 2011). MERRA and CFSR might have profited from their near-parallel execution, their close cooperation on input data, and early evaluations of the system (Saha et al. 2010; Rienecker et al. 2011). They are both based on the GSI (grid point statistical interpolation scheme, Kleist et al. 2009) implemented as 3D-Var, a joint development of the National Oceanic and Atmospheric Administration (NOAA) and NCEP, and the NASA and Global Modeling and Assimilation Office (GMAO). JCDAS are based on the 3D-Var method used at the JMA prior to February 2005 (Takeuchi and Tsuyuki 2002), and NCEP-R on the 3D-Var spectral statistical interpolation scheme operational at the NCEP in 1995 (Parrish and Derber 1992). Major advances in the more recent reanalyses also concern the observational input both in terms of quantity and quality. ERA-int, MERRA and CFSR perform direct assimilation of large quantities of satellite radiances, and apply automated variational schemes for correcting biases in the satellite radiances (Saha et al. 2010; Dee et al. 2011; Rienecker et al. 2011). This may improve their quality particularly over areas where conventional data are sparse, such as the tropics. Whereas the conventional observational input remained more or less steady over time, the quality of these observations is improved, e.g., with newly derived radiosonde temperature bias adjustments (Dee et al. 2011; Rienecker et al. 2011).

## Conclusions

### Results specific to the case study

We have not assessed whether skill and optimum scales of the reanalyses found in this study are transferable to regions outside the Cordillera Blanca, or to different variables. Here, we summarize important results confined to the assessed case study. In terms of air temperature predictors in the Cordillera Blanca, ERA-int show the highest skill of all considered reanalyses. Whereas CFSR and MERRA show comparably high skill like ERA-int, JCDAS and NCEP-R show considerably lower skill. More specifically, even if all reanalyses perform relatively well for wet-season months, differences in skill between the different reanalyses are evident especially during the dry-season months, and the intermediate-season months November and December. By using ensembles of all reanalyses, higher skill is obtained than by considering the reanalyses individually, despite ERA-int at their optimum domain.

Regarding the optimum scale analysis, ERA-int show the smallest optimum scale, with 2.88°. In the case of NCEP-R, most notably, the optimum scale is only twice the minimum scale. This implies the fewest amount of grid points to be averaged for NCEP-R, of all reanalyses. By contrast for MERRA and CFSR, the ratio between optimum scale and minimum scale is 14 and 13, respectively, and is thus the largest of all considered reanalyses. This result is somewhat surprising given that NCEP-R have the largest, and MERRA and CFSR the smallest minimum scale of the reanalyses. In terms of vertical levels, all reanalyses show the highest skill when data from pressure levels close to the study site are used, and vertical averaging hardly yields better results.

### General recommendations

Here we summarize conclusions to be generalized beyond the assessed case study. Even if optimum scales largely vary for different reanalyses, and the minimum scale is not necessarily a good indicator for the optimum scale (e.g., example of NCEP-R versus MERRA, CFSR), we generally recommend horizontal grid point averaging rather than using single grid points. Vertical averaging, by contrast, shows no significant increase in skill, and including data from pressure levels located distant from the study site lowers the skill considerably. The use of ensembles of reanalyses reduces errors even more efficiently than horizontal averaging, regardless of how many grid points of each reanalysis are included in the ensembles. Furthermore, we find that the more recent reanalyses with higher spatial resolutions and higher performance modelling systems and processing of observations (especially of satellite data) show notably higher skill than previous generation reanalyses. Finally, we like to point out that the analysis performed in this study can easily be repeated in different regions, or for other target variables, as long as a few-years observational data set is available. Because of the cross-validation procedure, the skill assessment is especially suited for short-term, high-resolution time series, with focus on inter-annual and intra-seasonal (day-to-day) variability.
